# Supplementary material for: Morphodynamic limits to environmental signal propagation across landscapes and into strata
Source: Nat Commun. 2022 Jan 12;13:292. doi: 10.1038/s41467-021-27776-6 (PMC8755758; doi:10.1038/s41467-021-27776-6)
Supplement: Supplementary file 1 — Supplementary Information [file 41467_2021_27776_MOESM1_ESM.pdf]

Supplementary information for *'Morphodynamic limits to environmental signal propagation across landscapes and into strata'*

by **Stephan C. Toby, Robert A. Duller, Silvio De Angelis, Kyle M. Straub**

Supplementary Note 1

The compensation timescale, T_c

The compensation timescale, T_c is an estimate of the maximum timescale at which autogenic processes can influence rates of surface elevation change through aggradation. At timescale >T_c *the* rate of surface elevation change is equal to the long term aggradation rate or tectonic subsidence, *r* (LT^{-1}). T_c also approximates the maximum time necessary to bury a particle to a depth that is no longer susceptible to erosion from a maximum, vertical topographic roughness length scale (l_{max}) at the Earth' surface[1,2,3]. Depending on the scale of sedimentological enquiry these roughness elements could be ripples, dunes, bars, channels[4,5]. The dynamics of these topographic roughness features define autogenic processes, and so T_c can be defined as:

$$T_c = \frac{l_{max}}{r} \qquad \text{(Eq. 1)}$$

and for channelized systems that we study here, where H_{max} represents the deepest channel:

$$T_c = \frac{H_{max}}{r} \qquad \text{(Eq. 2)}$$

The compensation timescale therefore, although developed for channelized systems, is applicable to any environment where topographic roughness features (ripples, dunes, bars, channels, etc.) migrate over a sediment surface that is net depositional over the long term, where 'long term' is not a single timescale but is dependent on the long term dynamics of the roughness features in question. For example in relatively quiescent lake and abyssal plain environments, the migration of roughness features like ripples, dunes, and bars will result in autogenic fluxes. These fluxes will saturate at some timescale T_c the value of which is commensurate with the physical scale and temporal dynamics of the autogenic system[5].

For the case of channelized system (Eq. 2), *Tc* represents an estimate of the maximum timescale of autogenic organization in stratigraphy[1,4,6].

Supplementary Note 2.

Defining the autogenic magnitude threshold or the autogenic threshold function

Toby *et al*., (2019)[7] constructed a theoretical framework to make predictions regarding the transfer of sediment flux signals to strata. T_c has been used as a singular temporal threshold for the transfer of relative sea level signals[8] and climate proxy signals[9] to the stratigraphic record. For example if the period of signal, S is greater than the compensation timescale, $S>T_c$ then the signal would be transferred to strata; but if $S<T_c$ then the signal would be shredded by autogenic processes that dominate at timescale less than T_c. Li *et al*. (2016)[8] incorporate signal amplitude into their framework using H_{max} at the shoreline to demarcate a minimum amplitude of sea level change required to be transferred to strata. Toby *et al*. (2019)[7] sought to understand by how much the sediment supply rate ("the signal") must vary in order to modify a landscape and store that signal in strata. The novel aspect of the theoretical framework of Toby *et al*. (2019)[7] was to recognize that at timescale less than T_c

the maximum rate of autogenic volume change (Q_A) decreases exponentially from Q_A at $T=0$ to Q_A at $T=T_c$. The implication of this is that sediment supply signals with periods less than T_c could still be transferred to strata, but only if the magnitude of the signal was greater than the associated Q_a for that particular period (or timescale) of signal. This is termed the autogenic threshold function (ATF) in the accompanying manuscript and is defined in figure 1a by the exponential line that marks the threshold between "signal shredding" and "signal transfer". The utility of this framework means that it is possible to predict the likely stratigraphic transfer of any combination of period and magnitude of a sediment supply signal. Toby *et al*. (2019)[7] validate this theoretical framework using laboratory experiments.

As a corollary to their work, Toby *et al*. (2019)[7] found that below the ATF in the signal shredding regime, some sediment supply signals induce a geomorphic response but not a stratigraphic response; and some produce neither a geomorphic or stratigraphic response. Toby *et al*. (2019)[7] show that the ability of sediment supply signals to produce a geomorphic surface response decreased as the period of sediment supply increased and defined a signal acceleration threshold to delineate these two different responses (diagonal dashed line, figure 1b). A rapid change in sediment supply, even if only small in total magnitude (such as a large flood), can trigger a transient geomorphic response at the Earth's surface, as the system cannot respond quickly enough to attain equilibrium with the sediment supply forcing. However it is not transferred to strata as the period of the sediment supply signal is short, and cannot produce a thick enough sedimentary pile to escape subsequent reworking by autogenic processes within the active surface layer. The region of the ATF in figure 1a defined as ESS (Earth surface signal) with diagonal fill represents the space where a geomorphic response or active layer response of this kind is likely. If the active layer reacts

to a sediment supply signal, or periodic sediment supply signal, then they are by definition more likely to convey the signal through the landscape or to the next segment in the sediment routing system, even though these signals are unlikely to be preserved in strata. It may indeed be the case that signals of this kind (i.e. those that occupy ESS space on fig. 1b) rapidly attenuate down-system, however depending on the initial magnitude of the signal it is possible that the signal could retain sufficient magnitude to plot above the ATF of the next segment, and therefore into strata. This of course relies on the ATF of the next segment to have a lower ATF threshold.

Supplementary Note 3.

Autogenic Threshold Function (ATF) in experiments

The autogenic threshold function (ATF) defined in Toby *et al.* (2019)[7] is calculated by measuring rates of autogenic volume changes for increasingly long time windows (Figure S1a). All volumetric sediment fluxes are made dimensionless by dividing by the long-term mean sediment supply rate (Q_{in}, e.g. $Q^*=Q/Q_{in}$). Timescales are made dimensionless by dividing time (t) by T_c ($T^*= t/T_c$; using an asterisk consistently for dimensionless variables). The maximum of these rates is Q_a^* $(=Q_a/Q_{in})$. The magnitude of Q_a^* decreases with (T^*) and is well described by an exponential function:

$$Q_a^* = Q_0^* e^{-bT^*} \qquad \text{(Eq. 3)}$$

where Q_0^* ($=Q_0/Qi_n$) is the intersect with the vertical axis (i.e. the maximum value of autogenic flux, Q_a^* at t=0) (Figure S1b) and parameter b is the decay constant of the exponential. We calculate this threshold function for three experiments (

) and find that decay of Q_a* with $T*$ is similar for each with the exception of experiment 3 (Figure S1b). At short timescales, experiment 3 shows much higher fluxes than the other two experiments. This is probably caused by autogenic scours that quickly eroded large volumes of sediment (Figure S1a). Erosion is limited in experiment 1 and 2 because of a cohesive polymer (see supplementary note 4 for information). We note that shorter timescales are also more sensitive to measurement noise, which may explain relatively large deviations between the empirical data and the exponential regression (Figure S1c).

	**Experiment 1**	**Experiment 2**	**Experiment 3**
**Dataset name**	**TDB-12 stage 2**	**TDB-13 stage 2**	**TDB-13 stage 1**
Run hours (hr)	385-1285	500-1000	75-300
Data source	(Li and Straub, 2017a)[10]	(Li and Straub, 2017b) [11]	(Li and Straub, 2017b) [11]
Cohesion	Strongly cohesive	Weakly cohesive	Non-cohesive
H_{max} (mm)	12.2	7.0	2.3
T_c (hr)	49	28	9
Q_{acc} (-)	0.53	0.66	0.73
Empirical equation	$Q_a* = 0.33\ e^{-0.49T*}$	$Q_a* = 0.46\ e^{-0.38T*}$	$Q_a* = 0.35\ e^{-0.18T*}$
Empirical Q_0*(-)	0.33	0.46	0.35
Field ATF-derived Q_0*(-)	0.47	0.34	0.27
Empirical b (-)	0.49	0.38	0.18
Field ATF-derived b (-)	≈3	≈3	≈3

Table S1. Information on laboratory experiments used to validate ATF-approach. Maintenance flux, $Q_{acc} = A{\cdot}r$ (where A is plan view area and r is sea level rise); compensation timescale, $T_c = H_{max}/r$; dimensionless maximum autogenic flux; $Q_0* = Q_0/Q_{in}$, where Q_0 is dimensional maximum autogenic flux at T = 0, and Q_{in} = long term mean sediment supply rate. See supplementary note 3 for explanation.

Supplementary Note 4.

Information on laboratory experiments used to validate the field Autogenic Threshold Function (ATF) approach

Laboratory experiments were conducted in the Tulane University Delta Basin, which is 4.2m long, 2.8m wide and 0.65m deep. Rate of sea level rise (as a proxy for uniform subsidence) is controlled to sub millimeter-scale resolution. Topography in all experiments was mapped once an hour with a FARO Focus3D-S 120 laser scanner on a 5-mm horizontal grid in the down and cross basin directions with a vertical resolution < 1 mm.

We make use of three stages of physical delta experiments[10,11] in the Tulane University Delta Basin (TDB). During each of these experimental stages an aggrading delta formed under constant conditions of sediment supply (3.91×10^{-4} kg/s), water discharge (1.72×10^{-4} m^3/s) and relative sea level (RSL) rise (0.25 mm/hr). In each experiment, the combination of sediment feed rate and base-level rise maintained the shoreline at an approximately constant location through the course of the experiment. The experimental stages all follow a similar layout, with one key difference between each of them: cohesion of the sediment mixture. Cohesion of the mixture is enhanced using a polymer that becomes adhesive when water is introduced[12,13]. See

for key information related to these experiments.

Experiment 1 represents run hours 385-1285 of experiment TDB-12-1[10], which made use of a strongly cohesive sediment mixture (1.47 g polymer per kg sediment). This mixture produced channelized deposits which formed delta lobes that avulsed by morphodynamic backwater effects[12]. Given maximum channel depth, H_{max} =12.2 mm and subsidence rate, r =

0.25 mm/hr, T_c is approximately 49 hr[14]. This experiment served as the control experiment to Toby *et al*. (2019)[7], and was used to construct the ATF threshold (figure 1b) of Toby *et al*. (2019)[7] and Figure S1b in the main document.

Experiment 2 included run hours 500-1000 of experiment TDB-13-1[11], which used a weakly cohesive sediment mixture (0.73 g polymer per kg sediment). Morphodynamic processes on this delta were similar to experiment 1, but T_c≈28 hr given that H_{max}=7 mm[14]. Experiment 3 made use of a non-cohesive sediment mixture with no polymer. This produced a semi-circular delta dominated by sheet flow, but interrupted approximately every 50 to 100 hr by deeper channels (Figure S1a).

Experiment 3 consisted of run hour 75-300 of experiment TDB-13-1[11]. We exclude the first 75 hr of experiment this experiment because the delta volume was low and in disequilibrium with forcing conditions (Figure S1a). H_{max}, given by the 95th percentile channel depth, is 2.3 mm and so it follows that T_c≈9 hr[14].

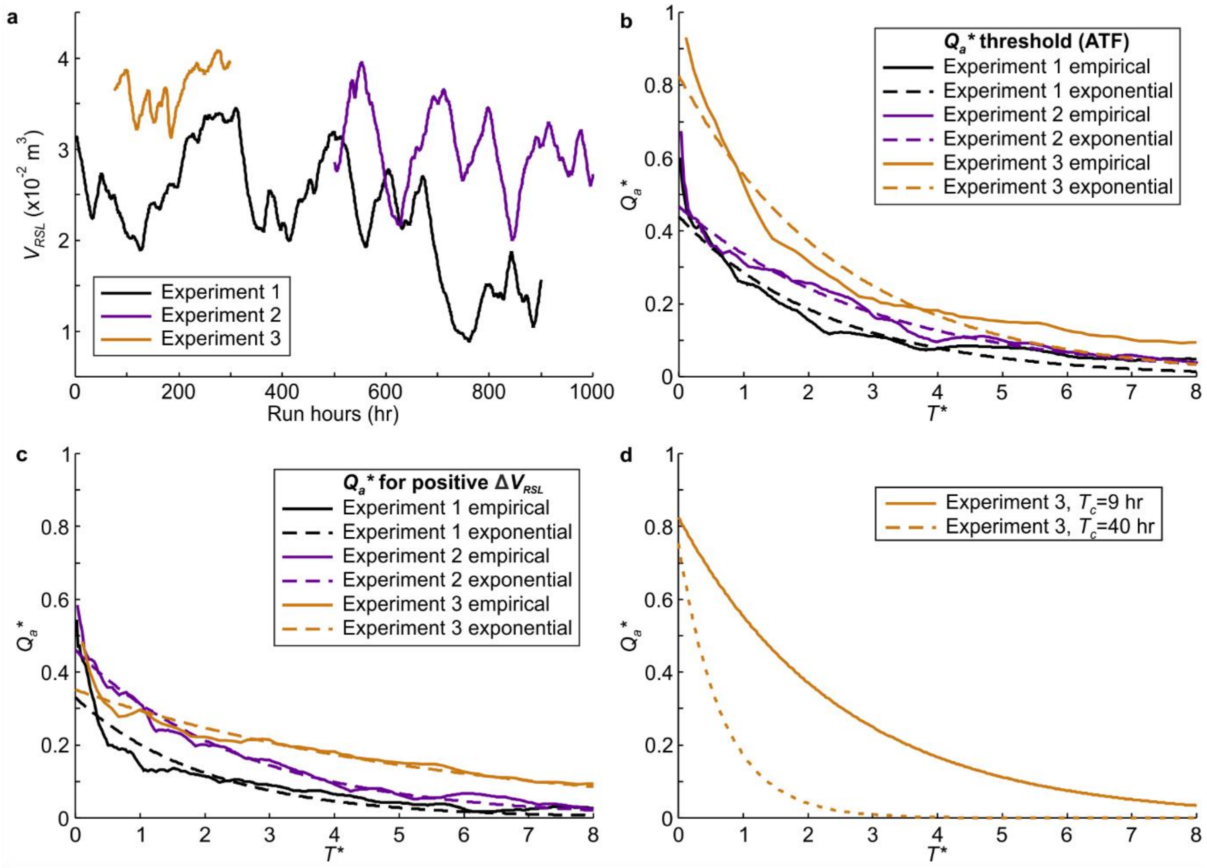

Figure S1. Calculation of Q_a* for each of the experiments. (a) Terrestrial volume (V_{RSL}) defined as the volume of sediment stored above sea level. These time series are used to calculate Q_a* following methods outlined in Toby *et al.* (2019)[7]. (b) Maximum absolute rate of terrestrial volume change measured from a high-resolution dataset (solid line), and an exponential fit through the data for time windows up to $8T_c$, which gives the autogenic threshold function (ATF). The equations are given in

and in supplementary note 3 and 4. Large-scale cycles in experiment 1 and 2 relate to lobe avulsions, but in experiment 3 these are generated by scouring channels. (c) Same as Figure S1b, but only using time windows of net positive terrestrial volume change. (d) Example of the Q_a* threshold in experiment 3 when a depth-estimate of the larger channels is used.

Supplementary Note 5.

Field approximation of the ATF

Recall that all volumetric sediment fluxes are made dimensionless by dividing them by the long-term mean sediment supply rate (Q_{in}, e.g. $Q^*=Q/Q_{in}$), and that timescales are made dimensionless by dividing time (t) by T_c ($T^*= t/T_c$; using an asterisk consistently for dimensionless variables). For an approximation of the ATF for field systems, parameters Q_0^* and b in equation 3 should be estimated from field data, which usually have low temporal and spatial resolution. For now, we focus on autogenic perturbations caused by excess sediment trapping. We approximate both parameters with a mass balance (Figure S2). Over long timescales, the mean allogenic sediment flux into the environment of interest (Q_{in}^*) is split in two parts: a maintenance flux (Q_{acc}^*) and bypass flux (Q_{bp}^*). Maintenance flux is the sediment flux necessary to balance the rate of accommodation generated by subsidence or eustatic sea level rise. For simplicity we assume a constant rate of accommodation generation. This means that Q_{acc}^* is constant and so the maintenance flux can be calculated from the plan-view area (A) and long-term aggradation rate: $Q_{acc}=A \cdot r$. The bypass flux, however, depends on the time window of measurement.

Over long-time windows (i.e. $t > T_c$), the rate of accommodation generation is equal to the rate of sedimentation (see supplementary note 1 above), as autogenic variations have leveled out at these long time windows. At $t \geq T_c$ this means $Q_a^* \approx 0$ and also that $Q_{bp}^*=Q_{in}^*-Q_{acc}^*$ and this provide the first data point (combination of Q_a^* and T^* at $T^* = t/T_c$) that can be used to define the field-ATF. However, over short time windows (i.e. $t<T_c$) $Q_a^*>0$ as autogenic processes dominate. The maximum autogenic flux occurs at very short time windows, where T^* approaches 0. A theoretical maximum to Q_a^* occurs when all sediment is trapped within an environment, and thus $Q_{bp}^*=0$. It follows that at $t \rightarrow 0$, $Q_0^*=Q_{in}^*-Q_{acc}^*$.

This maximum value of Qa* at T* = 0 provides an estimate of Q_0^*, and provides the second data point (combination of Qa* and T* at T* = t/T_c) that can be used to define the field-ATF.

Straub et al. (2015)[14] analyzed each of the experiments used here and calculated long-term mean sediment capture rates of the deltas (

), which we use as an estimate of Q_{acc}^*.

shows that our field approximation, given by $Q_0^*=Q_{in}^*-Q_{acc}^*$, gives a reasonable estimate to Q_0^* in the empirical exponential that uses positive volume changes only. The field ATF for experiment 1 is slightly lower than the empirical data, but this could be due to the general long-term negative trend in delta volume (Figure S1a) that reduces rates of positive volume change. Field estimates of Q_0^* for experiments 2 and 3 are slightly lower than the empirical functions suggest. The field estimates are based on average delta size, but higher rates of volume growth may occur at times where a delta is much smaller than the average size, leading to an underestimation of the threshold by our new field method. However, bearing in mind order of magnitude errors in sediment transport equations[15], our field methodology approximates Q_0^* reasonably well.

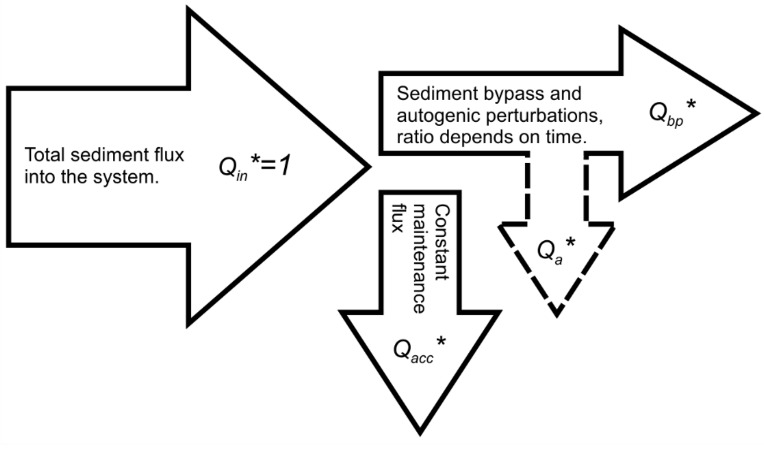

Figure S2. Schematic of sediment fluxes. Part of the allogenic sediment flux into an environment is used to keep up with accommodation generation (Q_{acc}^*). The other portion may bypass (Q_{bp}^*) or

generates perturbations on the long-term mean sedimentation rate (Q_a*). The ratio between Q_{bp}* and Q_a* depends on the time window of measurement.

After estimating Q_0*, parameter *b* is required to describe the exponential decay of the ATF. Any point on the ATF other than *T**=0 allows an estimation of *b*, and where field data allow this could be used to establish the ATF. In the absence of such data, we choose to approximate the timescale where the ATF approaches zero. Earlier we discussed that autogenic perturbations average out over long timescales. In an exponential function this is given by an asymptote with Q_a*=0. Inspired by the calculation of T_c as the 95th percentile channel depth to reduce the effects of rare extremes and measurement errors, we approximate a timescale (T_{95}) to reach the Q_a*-asymptote to within 5% of the maximum threshold value, Q_0*. In other words, T_{95} gives the point where the threshold value has reduced by 95% of the maximum value.

We use experiments 1 to 3 to investigate the timescale at which Q_a* asymptotes to within 0.05Q_0*. Even though the difference in cohesion between experiments generates very different morphodynamics, it appears that this timescale is approximately 6-8T_c for experiments 1-3 (Figure S1b). Theoretically, T_c approximates the maximum time window of stochastic autogenic processes[1], but there may still be an autogenic flux resulting from the averaging over multiple autogenic cycles. For example, avulsion timescales (T_A) in experiment 1 are of a timescale similar to T_c (Figure S1a) and so the variability in autogenic flux, due to these cycles of avulsion, must average out over timescales >>T_c, i.e. several channel depths worth of strata[6].

Most of the variability in terrestrial volume in experiment 1 and 2 relates to avulsion cycles, which are of a similar timescale as T_c. The timescale of avulsion cycles (T_A) in field-scale delta systems are often at least an order of magnitude shorter than T_c (see Table S2)

and therefore their effects level out over much shorter timescales, relative to T_c, in field-scale systems compared to laboratory experiments.

**Field-scale system**	**Avulsion timescale,** $\boldsymbol{T_A}$	**Compensation timescale,** $\boldsymbol{T_c}$	$\boldsymbol{T_A/T_c}$	**Information sources**
**Mississippi Delta**	1.4 x 10^3 yrs	200 x 10^3 yrs	0.007	T_A from Stouthamer and Berendsen (2007)[16]<br>T_c from Li *et al*. (2016)[8]
**Yellow River Delta**	0.6 x 10^3 yrs	30 x 10^3 yrs	0.002	T_A from Ganti *et al*. (2014)[17]<br>T_c from Li *et al*. (2016)[8]
**Yellow River (apex)**	0.007 x 10^3 yrs	2.06 x 10^3 yrs	0.018	T_A from Ganti *et al*. (2014)[17]<br>T_c from Chadwick et al. (2020)[18]
**Rhine River**	1.5 x 10^3 yrs	50 x 10^3 yrs	0.03	T_A from Stouthamer and Berendsen (2001)[19], Jerolmack and Mohrig (2007)[20]<br>T_c from Chadwick *et al*. (2020)[18], Stouthamer and Berendsen (2001)[19], Stouthammer et al. (2011)[21]
**Paraná River**	1.63 x 10^3 yrs	295 x 10^3 yrs	0.006	T_A from Chadwick *et al*. (2020)[18]<br>T_c from Zalan *et al*. (1990)[22] and Chadwick *et al*. (2020)[18]
**Orinoco River**	1.0 x 10^3 yrs	37 x 10^3 yrs	0.027	T_A from Chadwick *et al*. (2020)[18]<br>T_c from Li *et al*. (2016)[8]
**Po River**	1.0 x 10^3 yrs	17 x 10^3 yrs	0.06	T_A from Correggiari *et al*. (2005)[23]<br>T_c from Li *et al*. (2016)[8]

Table S2. Avulsion timescales (T_A) and compensation timescales (T_c) for field-scale systems.

Volume changes in experiment 3 are predominantly caused by autogenic oscillations between sheet flow and channel scouring. This suggests that even in systems where the largest autogenic processes are set by timescales of scouring, autogenic fluxes may exist at timescales several times longer than T_c. However, it should be noted that the volume changes in experiment 3 are mostly generated by channels with depths of 10 to 15 mm and occasionally even deeper, while H_{max}=2.3 mm was used to estimate T_c. This is because H_{max} is calculated as the 95th percentile channel depth, but it could be argued that the larger

channels set the depth of reworking, and thus T_c[1,24]. Using the deeper channels to estimate T_c would result in autogenic timescales that level out at timescales much closer to T_c. For example, a conservative H_{max}=10 mm for the larger channels in experiment 3 increases estimates of T_c to 40 hr. This effectively moves the dimensionless timescale at which autogenic processes level out much closer to T_c (Figure S1d). Therefore, the timescales for autogenic fluxes to level out may thus be much closer to T_c for field-scale systems where avulsion cycles are much smaller than T_c and the dominant autogenic scale is channel incision. Autogenic sediment fluxes occur at timescales less than T_c by definition, which means that T_c sets a minimum timescale for Q_a* to approach 0.

To approximate the threshold, we could now state that the timescale at which the ATF (i.e. Q_a*) reaches a value of $0.05Q_0$* is of the order of $1T_c$ (or 6-8T_c when $T_A \approx T_c$; but see discussion above on T_A/T_c for field-scale systems). Using Equation 3, we estimate b by approximating the point at which the ATF approaches 0 to within 5%:

$$0.05\, Q_0^* = Q_0^*\ e^{-bT_{95}^*} \qquad \text{(Eq. 4)}$$

$$b = -\ln(0.05)/T_{95}^* \qquad \text{(Eq. 5)}$$

where T_{95}* is T_{95} normalized by T_c. In a scenario where autogenic fluxes approach 0 in a time window of T_c, $b \approx 3$, while for the experiments T_{95}* is 6-8 times longer than T_c, and so this approximation suggests b is close to 0.4-0.5. To discuss further we note that Experiment 1 experienced long avulsion time scales only in the second half of the experiment (875-1285 hrs) as the delta was building into deep water, which inhibited in-channel aggradation and prolonged the avulsion timescale. Constructing the ATF for the first 490 hrs of experiment 1

shows that $0.05Q_0*$ is achieved at $1T_c$ and yields a b value that is closer to the predicted value of 3. We note that a better predictive understanding of the time windows at which long-term sedimentation rates persists in different environments will reduce uncertainty in approximating the ATF.

Supplementary Note 6.

Estimating the ATF using field data

To estimate the potential of the ESRS to store SFSs, we analyze a time slice between 39.1 and 36.5 Ma. Michael et al. (2014)[25] split the ESRS up into five segments and calculate sediment volumes preserved in the stratigraphy of the ESRS for each of those segments. From this, we estimate Q_{in} and Q_{acc} for each segment. Q_{acc} follows from the sediment volume preserved in a segment's stratigraphy. The sum of Q_{acc} for all segments gives an estimate of the total sediment flux from the system's catchment into the basin. Q_{in} for each segment is calculated by subtracting Q_{acc} from the Q_{in} of the previous segment. Therefore the bypass flux (Q_{bp}) of one segment is consequently the Q_{in} for the next segment. T_c is determined for each segment using measurements of channel depths and estimation of aggradation rate. Each ATF of the ESRS (Fig. 3 and Fig. 6) is accompanied by an error envelope that originates from the value ranges of Q_{in} and Q_{acc} (and so Q_{bp}). Errors on the ATF for the Gurb and Sis segments: $\pm 0.22Q_0*$, the Escanilla-Graus segment: $\pm 0.25Q_0*$ and the Escanilla-Ainsa segment $\pm 0.30Q_0*$. The ranges of field parameter values have also been propagated as errors to each data point on Figure 6. All errors can be reconstructed from table 2 in the manuscript.

References

1. Wang, Y. A., Straub, K. M. & Hajek, E. A. Scale-dependent compensational stacking: An estimate of autogenic time scales in channelized sedimentary deposits. *Geology* 39, 9, 811-814 (2011).
2. Straub, K. M. & Esposito, C.R. Influence of water and sediment supply on the stratigraphic record of alluvial fans and deltas: Process controls on stratigraphic completeness. *Journal of Geophysical Research - Earth Surface* 118, 1-13 (2013).
3. Straub, K. M. & Foreman, B.Z. Geomorphic stasis and spatiotemporal scales of stratigraphic completeness, *Geology 46*), 311-314 (2018).
4. Straub, K. M., Duller, R. A., Foreman, B. Z. & Hajek, E. A. Buffered, Incomplete, and Shredded: The Challenges of Reading an Imperfect Stratigraphic Record. *Journal of Geophysical Research-Earth Surface* 125, no. 3 (2020).
5. Ganti, V., Hajek, E. A., Leary, K., Straub, K. M. & Paola, C. Morphodynamic hierarchy and the fabric of the sedimentary record. Geophysical Research Letters 47, e2020GL087921. https://doi.org/ 10.1029/2020GL087921 (2020).
6. Sheets, B. A., Hickson, T. A. & Paola, C., 2002, Assembling the stratigraphic record: depositional patterns and time-scales in an experimental alluvial basin: Basin Research, v. 14, no. 3, p. 287-301.
7. Toby, S. C., Duller, R. A., De Angelis, S. & Straub, K. M. A stratigraphic framework for the preservation and shredding of environmental signals. *Geophysical Research Letters* 46, no. 11, 5837-5845 (2019).
8. Li, Q., Yu, L. Z. & Straub, K. M. Storage thresholds for relative sea-level signals in the stratigraphic record. *Geology* 44, no. 3, 179-182 (2016).
9. Foreman, B. Z. & Straub, K.M. Autogenic geomorphic processes determine the resolution and fidelity of terrestrial paleoclimate records. *Science advances* 3, no. 9, e1700683. (2017).
10. Li, Q. & Straub, K. M. TDB_12_1: SEAD (2017a).
11. Li, Q. & Straub, K. M. TDB_13_1: SEAD (2017b).
12. Hoyal, D. C. J. D. & Sheets, B. A. Morphodynamic evolution of experimental cohesive deltas. *Journal of Geophysical Research-Earth Surface* 114, F02009, doi:10.1029/2007JF000882 (2009).
13. Li, Q., Benson, W. M., Harlan, M., Robichaux, P., Sha, X. Y., Xu, K. H. & Straub, K. M. Influence of Sediment Cohesion on Deltaic Morphodynamics and Stratigraphy Over Basin-Filling Time Scales. *Journal of Geophysical Research-Earth Surface*, 122, no. 10, 1808-1826 (2017).
14. Straub, K. M., Li, Q. & Benson, M. Influence of sediment cohesion on deltaic shoreline dynamics and bulk sediment retention: A laboratory study. *Geophysical Research Letters*, 42, no. 22, 9808-9815 (2015).
15. Ma, H. B., Nittrouer, J. A., Naito, K., Fu, X. D., Zhang, Y. F., Moodie, A. J., Wang, Y. J., Wu, B. S. & Parker, G. The exceptional sediment load of fine-grained dispersal systems: Example of the Yellow River, China: Science Advances 3, e1603114 (2017).
16. Stouthamer, E., and Berendsen, H. J. A., 2007, Avulsion: The relative roles of autogenic and allogenic processes: Sedimentary Geology, v. 198, no. 3-4, p. 309-325.
17. Ganti, V., Chu, Z., Lamb, M.P., Nittrouer, J.A. & Parker, G. Testing morphodynamic controls on the location and frequency of river avulsions on fans versus deltas: Huanghe (Yellow River), China. *Geophys. Res. Lett*. 41, 7882-7890 (2014).
18. Chadwick, A.J., Lamb, M.P. & Ganti, V. Accelerated river avulsion frequency on lowland deltas due to sea-level rise. *PNAS* 117, no. 30, 17584-17590 (2020).

19. StouthammerE. & Berendsen, H.J.A. Avulsion frequency, avulsion duration and interavulsion period of Holocene channel belts in the Rhine-Meuse delta, The Netherlands. *Journal of Sedimentary Research* 71, 588-597 (2001).

20. Jerolmack, D. J. & Mohrig, D. Conditions for branching in depositional rivers. *Geology* 35, no. 5, 463-466 (2007)

21. Stouthammer, E., Cohen, K.M., and Gouw, M.J.P. Avulsion and its implications for fluvial deltaic architecture: insights from the Holocene Rhine-Meuse Delta. in From River To Rock Record: The Preservation Of Fluvial Sediments And Their Subsequent interpretation. (eds. Davidson, S.K., Leleu, S., and North, C.) SEPM Special Publication No. 97, 215-231 (2011).

22. Zalan, P.V., Wolff, S., Astolfi, M.A.M., Vieira, 1.S., Concelcao, J.C.J., Appi, V.T., Neto, E.V.S., Cerqueira, J.R.& Marques, A. The parana basin, Brazil, in Cratonic Basins 619 interior cratonic basins (Eds. By Leighton, M.W., Kolata, D.R., Oltz, D., and Eidel, J.J.), 681–708, American Association Petroleum Geologists Memoir 51 (1990).

23. Correggiari, A., Cattaneo, A. & Trincardi, F. Depositional Patterns in the Late Holocene Po Delta System. in River Deltas - Concepts, Models and Examples (eds. Bhattacharya, J.P. and Giosan, L.) SEPM Special Publication No. 83, 365-392 (2005).

24. Straub, K. M., Paola, C., Mohrig, D., Wolinsky, M.A. & George, T. Compensational stacking of channelized sedimentary deposits. *Journal of Sedimentary Research*, 79, No. 9, 673-688 (2009).

25. Michael, N. A., Whittaker, A. C., Carter, A. & Allen, P. A. Volumetric budget and grain-size fractionation of a geological sediment routing system: Eocene Escanilla Formation, south-central Pyrenees. Geological Society of America Bulletin 126, no. 3-4, 585-599 (2014).
